# Group therapy in Schizophrenia. What’s the evidence?

**DOI:** 10.1192/j.eurpsy.2022.1924

**Published:** 2022-09-01

**Authors:** F. Azevedo, L. Silva, A.A. Quintão, N. Moura, P. Duarte

**Affiliations:** 1 Centro Hospitalar de Lisboa Ocidental, Psychiatry, Lisbon, Portugal; 2 Unidade Local de Saúde do Baixo Alentejo, Psychiatry, Beja, Portugal; 3 Centro Hospitalar de Lisboa Ocidental, Psychiatry Department, Lisboa, Portugal; 4 Centro Hospitalar Lisboa Ocidental, Psychiatry, Lisbon, Portugal

**Keywords:** psychosocial, schizophrénia, GROUP THERAPY

## Abstract

**Introduction:**

The American Psychiatric Association and NICE’s Guidelines for schizophrenia recommend psychosocial interventions as adjuvants to pharmacological treatment, highlighting the role of cognitive behavioral therapy for psychosis, psychoeducation, family intervention, cognitive remediation, autonomy training, social skills training, and supported employment. Although highly recommended in their individual forms current guidelines make no definitive statement about their group applicability.

**Objectives:**

The goal of this work was to critically review the evidence of group interventions in schizophrenia

**Methods:**

Non-systematic review of the literature with selection of scientific articles published in the past 10 years; by searching Pubmed and Medscape databases using the combination of MeSH descriptors. The following MeSH terms were used: “schizophrenia”, “group therapy”.

**Results:**

Group therapy has shown important benefits in different conditions over the years, likely through mechanisms such as peer motivation, controlled confrontation, increased insight and even a tendency to homogenous results between group participants through peer influence. These results have been reproduced in schizophrenia though the benefits of applying group concepts to structured psychosocial interventions is still under study.

**Conclusions:**

Recent evidence suggests some evidence-based interventions can be applicable in group form, namely social skills training, cognitive remediation, psychoeducation, and multifamily groups, synergizing the already known benefits with newer therapy models and decreasing costs for patients and healthcare systems. Adequate controlled studies between individual and group therapy will shed further light on this matter.

**Disclosure:**

No significant relationships.

